# Treatment of Fibrous Tumors of the Uterus by Ergot

**Published:** 1875-07-01

**Authors:** W. H. Byford


### TREATMENT OF FIBROUS TUMORS OF THE UTERUS BY ERGOT.

Abstract of a Paper read at the Meeting of the American Medical Association, in Louisville, May, 1875, by W. H. Byford, M. D.

THE publication of Hildebrandt's articles on the use of ergot in the treatment of fibrous tumors of the uterus, had for its object the solution of the question, viz.: will ergot effect a cure of these tumors?

The analysis of one hundred and three cases, the histories of which

I have obtained from journals and correspondents, answers the question conclusively, as I think, in the affirmative. Twenty-three cases out of the whole number are reported cured; in thirty-eight more the tumors were diminished in size, and the hæmorrhage and other disagreeable symptoms removed; nineteen of the remainder were benefited by the relief of the hæmorrhages and leucorrhœal discharges, while the size and other conditions of the tumors were unchanged. Of the total number, only twenty-one entirely resisted treatment. This shows results decidedly favorable in eighty-two of the hundred and three cases. We may still further appreciate the favorable effects of the treatment, by the consideration that in twenty-one cases it was suspended, which is as great a number as resisted treatment. It is a noticeable fact that some of the cases in which the treatment was suspended, were very much benefited by it. The great obstacle to arriving at accurate results, has been the difficulty in carrying out the treatment. Not much uniformity has been observed in the manner of using the ergot. Some recommend and use it hypodermically only, while others administer it hypodermically, internally by the stomach, and in the form of suppositories in the vagina and rectum. The principal objections to the use of the hypodermic method are, the pain inflicted by the needle, and the inflammation and suppuration which ensue in a large proportion of cases. On this account many patients who began treatment refused to continue it, and their cases were abandoned. Where there has not been too much exhaustion, or too great gastric irritability, ergot has been given internally with beneficial results in a majority of instances, while in a few it seemed to have no influence whatever, where marked benefit had been observed when it was given hypodermically.

There has been as little uniformity in selecting the place at which to make the injection as there has been in the method of administering the remedy. The deltoid region, just posterior to the great trochanter, and the lower part of the abdomen have been the principal places selected, but it undoubtedly makes but little difference where the insertion is made. Several cases have been reported where the injections have been made into the cervix uteri and the substance of the tumor, when accessible, with very beneficial results.

As the preparation of the medicine employed seems to have had much to do in causing the irritation, especially when given hypodermically, efforts have been made to find some form that would not produce the inflammation so often resulting in abscesses. Hildebrandt is now in the habit of using Dr. Wernich's formula for the watery extract of ergot, which according to Dr. Mundi is very similar to the solid extract of ergot made by Dr. Squibb. Most American practitioners now use Dr. Squibb's solid extract; some of them by dissolving in pure water, while others add to the water a small amount of pure glycerine. Dr. Squibb recommends a solution of this extract to be made as follows: Dissolve 200 grains of the extract in 250 minims of water, by stirring; filter the solution through paper, and make up to 300 minims by washing the residue on the filter with a little water. Each minim of this solution represents four grains of the ergot in powder. Of this solution, from ten to twenty minims are injected once a day or once in two days. This is the only preparation I have used in hypodermic injections, and I believe it is the best we can at present procure.

There is undoubtedly great necessity in having the solution freshly prepared, as in a very short time it deteriorates and becomes more irritating to the tissues. When ergot is administered freshly prepared, it generally produces prompt effects. In most instances in half an hour the patient experiences painful contractions of the uterus. The hand applied over the organ at once recognizes the increased hardness in the mass. These contractions increase in severity for the first two hours and then continue with vigor from six to ten hours, gradually becoming less until they cease entirely. Some patients suffer so much from these pains as to refuse to proceed in the treatment, while others bear them without much inconvenience. We do not always observe these painful effects, even when the drug operates very beneficially. Sometimes the hæmorrhages are controlled as it were insensibly, and the tumor slowly decreases in size without the patient experiencing any considerable discomfort. It seems highly probable from the statements made by my correspondents, as well as from my own observations, that the benefits of the remedy are produced with more rapidity in the early part of the treatment.

The preparation used internally more frequently than any other, is the fluid extract, either alone or in combination with belladonna. Each minim of Squibb's fluid extract is equal to one grain of ergot. Some recommend it to be given in doses of thirty drops, three or four times a day. Others believe it should be given in larger doses, less frequently repeated, as per example: one drachm once or twice in twenty-four hours. It is efficacious given in either way, but probably more so in the larger and less frequent doses. This preparation is so offensive and causes so much nausea in exceptional instances that it cannot be borne. Dr. Squibb claims that his solid extract does not offend the stomach so frequently as the fluid extract. The solid extract may be used in pills coated with gelatine. A pill of five grains is equal to thirty grains of crude ergot, and may be administered twice or three times daily.

From observation of the effects of the different preparations, I am satisfied that this is altogether the most efficient and agreeable for internal administration. A suppository for the rectum may be composed of fifteen grains of the solid extract and enough gelatine to give it size and form. I have no doubt of the great usefulness of this method of administering ergot. I think it is also quite certain that the addition of belladonna in some cases increases the curative effects of ergot, but to what extent it is difficult to ascertain. Ergot produces many good effects beside reducing the size of the tumor and affording relief of the hæmorrhages. I have seen, and some of my correspondents mention, great functional improvement in the more important organs. Some patients are relieved by it of obstinate constipation; the appetite is improved and the general health restored. This remarkable effect is obviously due to its action on the ganglionic nervous system. In exceptional instances ergot produces very disagreeable effects, such as great heat and tenderness in the uterine region, metritis, phlebitis, vertigo, nervous perturbation, etc.

The beneficial effects of ergot may be increased by the means of auxiliary treatment. The well known alterative and sorbefacient medicines have in rare instances been credited with the cure of tumors without the aid of ergot, and it is not difficult to understand that absorption may be promoted with more certainty by the alkaline bromides and iodides, where the vitality of the tumor is first impaired by the action of ergot on its vessels and the muscular fibres surrounding it. A few, who have reported cases cured, have prescribed as auxiliary treatment the iodide of potassium and the bichloride of mercury, and quite a number have combined belladonna with the ergot. How much may be effected by judicious alterative and auxiliary treatment will doubtless be determined by future observation.

Much can be done to prevent or ameliorate the disagreeable effects of ergot in certain exceptional instances. The distressing pain caused by it may sometimes be made more tolerable by the administration of hydrate of chloral, without very materially influencing its other effects. Indigestion, constipation, and nervous debility may be corrected by tonics, alteratives, laxatives, and stimulants given at the same time with ergot. In short, the general condition of the patient should be cared for in the same rational manner as if ergot was not being administered.

Observation seems to show that a fibrous tumor of the uterus may be affected by ergot in three ways:

1st. It is gradually disintegrated and absorbed. In this way it disappears without any violent or disagreeable symptoms.

2d. Its nutrition is so interrupted as to produce a rapid destruction of its vitality; and hence decomposition within the capsule and a semi-putrid mass expelled. This process is accompanied with evidences of inflammation of the uterus and toxæmia, more or less grave, according to the size of the tumor, the length of time between the commencement of decomposition and the expulsion of the tumor, and the vital resistance of the patient.

3d. The tumor in nearly its original condition is totally or partially expelled from the cavity of the uterus, attended with varying degrees of inversion of the organ. In this condition it becomes amenable to surgical processes for completing its removal.

